# Whey Protein Concentrate WPC-80 Intensifies Glycoconjugate Catabolism and Induces Oxidative Stress in the Liver of Rats

**DOI:** 10.3390/nu10091178

**Published:** 2018-08-28

**Authors:** Marta Żebrowska-Gamdzyk, Mateusz Maciejczyk, Anna Zalewska, Katarzyna Guzińska-Ustymowicz, Anna Tokajuk, Halina Car

**Affiliations:** 1 Lomza State University of Applied Sciences, 14 Akademicka Street, 18-400 Lomza, Poland; 2 Department of Experimental Pharmacology, Medical University of Bialystok, 37 Szpitalna Street, 15-767 Bialystok, Poland; ania.tokajuk@gmail.com (A.T.); halina.car@umb.edu.pl (H.C.); 3 Department of Physiology, Medical University of Bialystok, 2c Mickiewicza Street, 15-233 Bialystok, Poland; 4 Department of Conservative Dentistry, Medical University of Bialystok, 24a M. Sklodowskiej-Curie Street, 15-274 Bialystok, Poland; azalewska426@gmail.com; 5 Department of General Pathomorphology, Medical University of Bialystok, 24a M. Sklodowskiej-Curie Street, 15-274 Bialystok, Poland; kguzinska74@gmail.com

**Keywords:** exoglycosidases, liver, oxidative stress, whey

## Abstract

The aim of this study was to evaluate the effect of whey protein concentrate (WPC-80) on glycoconjugate catabolism, selected markers of oxidative stress and liver inflammation. The experiment was conducted on male Wistar rats (*n* = 63). The animals from the study group were administered WPC-80 at a dose of 0.3 or 0.5 g/kg body weight for 7, 14 or 21 days, while rats from the control group received only 0.9% NaCl. In liver homogenates, we assayed the activity of N-acetyl-β-D-hexosaminidase (HEX), β-glucuronidase (GLU), β-galactosidase (GAL), α-mannosidase (MAN), α-fucosidase (FUC), as well as the level of reduced glutathione (GSH), malondialdehyde (MDA), interleukin-1β (IL-1β) and transforming growth factor-β1 (TGF-β1). A significantly higher activity of HEX, GLU, MAN and FUC were found in the livers of rats receiving WPC-80 compared to controls. Serum ALT and AST were significantly higher in the animals supplemented with WPC-80 at a dose of 0.5 g/kg body weight for 21 days. In the same group of animals, enhanced level of GSH, MDA, IL-1β and TGF-β1 were also observed. WPC-80 is responsible for intensive remodelling of liver tissue and induction of oxidative stress especially at a dose of 0.5 g/kg body weight.

## 1. Introduction

In recent years, there has been an increase in interest in whey [1]. It is a by-product of cheese production and a rich source of exogenous amino acids and biologically active proteins. It has been proven that α-lactalbumin and β-lactoglobulin are the main whey proteins, forming up to 80% of the protein mass [2,3]. Other whey peptides include: immunoglobulins, albumins, lactoferrin and lactoperoxidase [3]. Due to its varied composition, whey is commonly supplemented to emaciated patients (e.g., during convalescence or cancer cachexia), children with cow’s milk protein allergy and sportsmen to increase their muscle mass [1,4]. Whey proteins are also a valuable source of sulphuric amino acids: cysteine and methionine, crucial for the synthesis of reduced glutathione (GSH) [5]. GSH reveals strong antioxidant properties, which affects the proper functioning of the body, both healthy and ill [5].

Excess protein in the diet (including whey proteins) can adversely affect the activity of the organs participating in its metabolism [6]. One of such organs is the liver. It has been demonstrated that high-protein diet may result in a positive nitrogen balance, which causes increased production of urea and ammonia in the urea cycle [7]. This situation may lead to a significant overload of the liver [6,8]. One of the markers used to assess liver function are lysosomal exoglycosidases: N-acetyl-β-D-hexosaminidase (HEX, EC 3.2.1.52), β-glucuronidase (GLU, EC 3.2.1.31), β-galactosidase (GAL, EC 3.2.1.23), α-mannosidase (MAN, EC 3.2.1.24) and α-fucosidase (FUC, EC 3.2.1.51) [9,10,11]. These enzymes are responsible for the degradation of individual monosaccharide residues from the non-reducing end in the oligosaccharide chain of glycoconjugates [12].

Increased protein supply in the diet (above the current needs of the body) may also induce oxidative stress [13]. This process involves increased production of reactive oxygen species (ROS) and impairment of enzymatic and non-enzymatic antioxidant mechanisms [14]. As a result, oxidative stress disrupts cell metabolism and can even lead to cell death by apoptosis or necrosis [15]. Lipids are the first to suffer oxidative damage, as they are the most susceptible to oxygen free radicals [16,17]. Although increased production of ROS under the influence of rich protein diet has been demonstrated in numerous organs (brain [18], pancreas [19], salivary glands [20] and liver [6]), the effect of whey on liver oxidative damage is still unknown. Whey reveals documented antioxidant properties [21] but it has not been established yet whether whey-induced boost in GSH synthesis can prevent oxidative stress in the conditions of increased protein supply. The existing literature on the subject also lacks data on the effect of whey protein concentrate (WPC-80) on liver function. Therefore, the aim of this study was to evaluate the activity of lysosomal exoglycosidases and selected markers of oxidative stress and inflammation of liver homogenates in rats fed with whey protein concentrate WPC-80.

## 2. Materials and Methods

The experiment had been approved by the Local Ethical Committee on Animal Testing: No. 106/2015 (Medical University of Bialystok, Poland).

### 2.1. WPC-80 Composition

Whey protein concentrate used in the study was produced by Dairy Cooperative in Mońki, Poland. WPC-80 was analysed in the accredited laboratory at SJ Hamilton Poland LTD (Gdynia, Poland) and Rtech laboratory at Land O’Lakes Laboratories (St. Paul, MN, USA). The content of proteins, carbohydrates, fat, ash, dietary fibre, amino acids, fatty acids, vitamins, as well as minerals was assayed. The humidity and calorific values of WPC-80 were also evaluated.

### 2.2. Animals

The experiment was conducted on 6–7-week-old outbreed male Wistar rats with an initial body weight of 180–250 g. The animals were provided with 12-h light/dark cycle, constant air temperature (20–21 °C ± 2 °C) and unlimited access to food (standard granulated food for rats: Agropol, Motycz, Poland; 10.3 kcal% fat, 24.2 kcal% protein, 65.5 kcal% carbohydrates) and drinking water. Upon arrival at the animal quarters and after 1 week of adaptation to the new surroundings, the animals were randomly divided into 9 groups of 7 (*n* = 7):

1. C7—control group receiving 0.9% NaCl for 7 days
2. C14—control group receiving 0.9% NaCl for 14 days
3. C21—control group receiving 0.9% NaCl for 21 days
4. 0.3 WPC 7—a group receiving WPC-80 at a dose of 0.3 g/kg body weight for 7 days
5. 0.3 WPC 14—a group receiving WPC-80 at a dose of 0.3 g/kg body weight for 14 days
6. 0.3 WPC 21—a group receiving WPC-80 at a dose of 0.3 g/kg body weight for 21 days
7. 0.5 WPC 7—a group receiving WPC-80 at a dose of 0.5 g/kg body weight for 7 days
8. 0.5 WPC 14—a group receiving WPC-80 at a dose of 0.5 g/kg body weight for 14 days
9. 0.5 WPC 21—a group receiving WPC-80 at a dose of 0.5 g/kg body weight for 21 days

The dose of WPC-80 was selected on the basis of the literature analysis [3,22,23]. Immediately prior to administration, an appropriate amount of WPC-80 was dissolved in 0.9% solution of NaCl (saline solution). WPC-80 was administered intragastrically every day, always at the same time, in the volume of 2 mL/kg body weight. At the same time, rats from the control group received saline solution intragastrically in the amount of 2 mL/kg body weight [3].

After 7, 14 or 21 days, sodium pentobarbital was administered intraperitoneally at a dose of 45 mg/kg body weight to rats of the respective groups (before pentobarbital administration, rats were denied food for 12 h). The rats had abdominal aortic blood collected and then their tissues were collected for further examination. The liver tissue was rinsed with cold phosphate buffered saline (PBS; 0.02 M, pH 7.4) and dried, then frozen in liquid nitrogen and stored at −80 °C until the day of assay. The liver specimen was also fixed with 10% buffered formalin.

The blood was collected for a test tube with EDTA (ethylenediaminetetraacetic acid) and a test tube with a clot activator. Complete blood counts (WBC, leukocytes; RBC, erythrocytes; HCT, haematocrit; HGB, haemoglobin; MCV, Mean Corpuscular Volume; MCH, Mean Corpuscular Haemoglobin; MCHC, Mean Corpuscular Haemoglobin Concentration; PLT, platelets) were analysed in the whole blood. Immediately after centrifugation (10 min, 1500× *g*, 4 °C; MPW-351, Mechanika Precyzyjna S.C., Warsaw, Poland), the obtained serum was tested for biochemical parameters (ALT, alanine aminotransferase; AST, aspartate aminotransferase; bilirubin; albumin; Cr, creatinine; UA, uric acid; urea; TC, total cholesterol). All determinations were performed using ABX Pentra 400 (Horiba, Northampton, UK). Plasma glucose level was determined using colorimetric kit. The liver index was calculated using the formula: liver index = liver weight/body weight *×* 100%.

### 2.3. Preparation of Homogenates

On the day of assays, the livers were slowly thawed in 4 °C, weighed and divided into two equal parts, one of which was diluted in 0.15 M potassium chloride solution with 0.2% Triton X-100 at a ratio of 1:10 (i.e., 1 g of tissue per 1 mL of solvent) to assess the activity of lysosomal exoglycosidases [12]. The other part of the tissue material was diluted in 0.02 M phosphate-buffered saline (pH 7.4) at a ratio of 1:10—to evaluate the concentration of total protein, reduced glutathione and malondialdehyde [24]. In order to prevent sample oxidation and proteolysis, butylated hydroxytoluene (BHT; 10 μL 0.5 M BHT in acetonitrile/1 mL buffer) and proteolysis inhibitor (1 tablet/10 mL buffer; Complete Mini Roche, France) were added [25,26]. The obtained tissue suspensions were homogenized with a glass homogenizer (Omni TH, Omni International, Kennesaw, GA, USA) and then centrifuged (30 min, 12,000× *g*, 4 °C). For further studies, a supernatant fluid was retained to be used immediately to perform the assays.

### 2.4. Evaluation of the Activity of Lysosomal Exoglycosidases

The activity of the selected lysosomal exoglycosidases (HEX, GLU, GAL, MAN, FUC) was determined by colorimetric micro methods. All assays were performed in triplicate samples and standardized to 100 mg of total protein. Absorbance was measured with the microplate reader Mindray MR-96-A, Hamburg, Germany.

#### 2.4.1. Evaluation of HEX Activity

The activity of HEX was evaluated according to the method described by Marciniak et al. [27]. 40 μL citrate-phosphate buffer (0.1 M, pH 4.7) and 30 μL 20 mM substrate solution (p-nitrophenyl-2-acetamido-2-deoxy-β-d-glucopyranoside, Sigma-Aldrich, Steinheim, Germany) were added to 10 μL supernatant fluid. The reaction mixture was incubated on a shaker (DTS-4 Sky-Line, Elmi, Riga, Latvia) for 60 min at 37 °C. The reaction was stopped by adding 200 μL borate buffer (0.2 M, pH 9.8). The absorbance of the released p-NP was measured at 405 nm wavelength.

#### 2.4.2. Evaluation of GLU Activity

GLU activity was determined by the Marciniak et al. [27] method. 40 μL acetate buffer (0.1 M, pH 4.5) and 30 μL 20 mM substrate solution (p-nitrophenyl-β-d-glucopyranoside, Sigma-Aldrich; Steinheim, Germany) were added to 10 μL supernatant fluid. The reaction mixture was incubated on a shaker for 60 min at 37 °C. The reaction was stopped by adding 200 μL borate buffer (0.2 M, pH 9.8). The absorbance of the released p-NP was measured at 405 nm.

#### 2.4.3. Evaluation of GAL Activity

The activity of GAL was determined according to the method described by Chojnowska et al. [28]. 40 μL citrate-phosphate buffer (0.1 M, pH 4.3) and 30 μL 1.6 mM substrate solution (p-nitrophenyl-β-d-galactopyranoside, Sigma-Aldrich; Steinheim, Germany) were added to 10 μL supernatant fluid. The reaction mixture was incubated on a shaker for 60 min at 37 °C. The reaction was stopped by adding 200 μL borate buffer (0.2 M, pH 9.8). The absorbance of the released p-NP was measured at 405 nm wavelength.

#### 2.4.4. Evaluation of MAN Activity

MAN activity was determined by Chojnowska et al. [28] method. 40 μL citrate-phosphate buffer (0.1 M, pH 4.3) and 30 μL 0.8 mM substrate solution (p-nitrophenyl-α-mannopyranoside, Sigma-Aldrich; Steinheim, Germany) were added to 10 μL supernatant fluid. The reaction mixture was incubated on a shaker for 60 min at 37 °C. The reaction was stopped by adding 200 μL borate buffer (0.2 M, pH 9.8). The absorbance of the released p-NP was measured at 405 nm.

#### 2.4.5. Evaluation of FUC Activity

FUC activity was determined by Chojnowska et al. [28] method. 40 μL citrate-phosphate buffer (0.1 M, pH 4.3) and 30 μL 2.3 mM substrate solution (p-nitro-α-fucopyranoside, Sigma-Aldrich; Steinheim, Germany) were added to 10 μL supernatant fluid. The reaction mixture was incubated on a shaker for 60 min at 37 °C. The reaction was stopped by adding 200 μL borate buffer (0.2 M, pH 9.8). The absorbance of the released p-NP was measured at 405 nm wavelength.

### 2.5. Evaluation of Oxidative Stress Markers

The concentrations of reduced glutathione (GSH) and lipid peroxidation products (malondialdehyde, MDA) were determined using the colorimetric micro methods. All assays were performed in triplicate samples and standardized to 100 mg of total protein.

#### 2.5.1. Evaluation of GSH Concentration

GSH concentration was assayed by the Ellman method using 5,5′-dithiobis-2-nitrobenzoic acid (DTNB) [29]. 100 μL 10% TCA and 100 μL 10 mM EDTA were added to 100 μL of supernatant. The samples were placed in a refrigerator (4 °C, 10 min) and then centrifuged for 5 min (5000× *g*, 4 °C). Next, 20 μL of deproteinised supernatant was collected and 180 μL of distilled water, 15 μL of 10 mM EDTA, 20 μL of 3.2 M TRIS buffer from HCl at pH = 8.1 and 10 μL DTNB were added. The absorbance of the supernatant was measured at 412 nm wavelength.

#### 2.5.2. Evaluation of MDA Concentration

The concentration of MDA was measured with the colorimetric method using thiobarbituric acid (TBA) [30]. 250 μL distilled water, 500 μL 15% trichloroacetic acid (TCA) and 500 μL 0.37% TBA were added to 250 μL of the sample. The samples were water bath-heated for 10 min and then centrifuged for 10 min (10,000× *g*, 4 °C). The absorbance of the supernatant was measured at 535 nm wavelength.

### 2.6. Assessment of Pro-Inflammatory Cytokines

Levels of pro-inflammatory cytokines, IL-1β (interleukin-1β) and TGF-β1 (transforming growth factor), were determined by ELISA method using ready-made kits (R&D Systems, Canada, Minneapolis, MN, USA). The absorbance was measured at 450 nm. All assays were performed in duplicate samples and standardized to 100 mg of total protein.

### 2.7. Assessment of Total Protein Concentration

Total protein concentration was determined by means of the commercial BCA^TM^ Protein Assay Kit (Pierce, Thermo Fisher Scientific, Rockfold, IL, USA). The principle of the method used is the reaction of Cu^+^ ions (formed upon the reduction of Cu^2+^ copper ions by protein molecules in the alkaline environment) with BCA bicinchoninic acid, resulting in a purple complex with a maximum absorbance at 562 nm wavelength.

### 2.8. Histological Examination

Histopathological examinations were performed by an experienced pathologist (K. G. U.). The liver sections were stained with haematoxylin and eosin (H&E) and examined under a light microscope (OPLYMPUS BX 51-P, OLYMPUS, Center Valley, PA, USA) at 10×, 20× and 40× magnification.

### 2.9. Statistical Analysis

The statistical analysis of the obtained results was performed using Statistica 12.0 statistical package (StatSoft, Tulsa, OK, USA) and GraphPad Prism 5.0 (GraphPad Software, Inc., La Jolla, CA, USA), with nonparametric tests: the ANOVA Kruskal-Wallis test and the Mann-Whitney test. Multiplicity adjusted *p* value was also calculated. The relationship between the quantitative data was assessed according to Spearman rank correlation. The results are presented as a median, minimum, maximum and percentiles. The threshold for statistical significance was *p* < 0.05. The sample size was set based on a previously conducted pilot study. The power of the test was set at 0.9.

## 3. Results

### 3.1. WPC-80 Composition

According to the analyses, the main component of WPC-80 is protein, which accounts for 78.2% of the concentrate mass. The preparation also contains fats, carbohydrates and small amounts of dietary fibre (Table 1). The remaining components of WPC-80 (endogenous and exogenous amino acids, fatty acids, vitamins and minerals) are listed in the Supplementary Material.

### 3.2. Characteristics of the Animals

**Figure 1:**
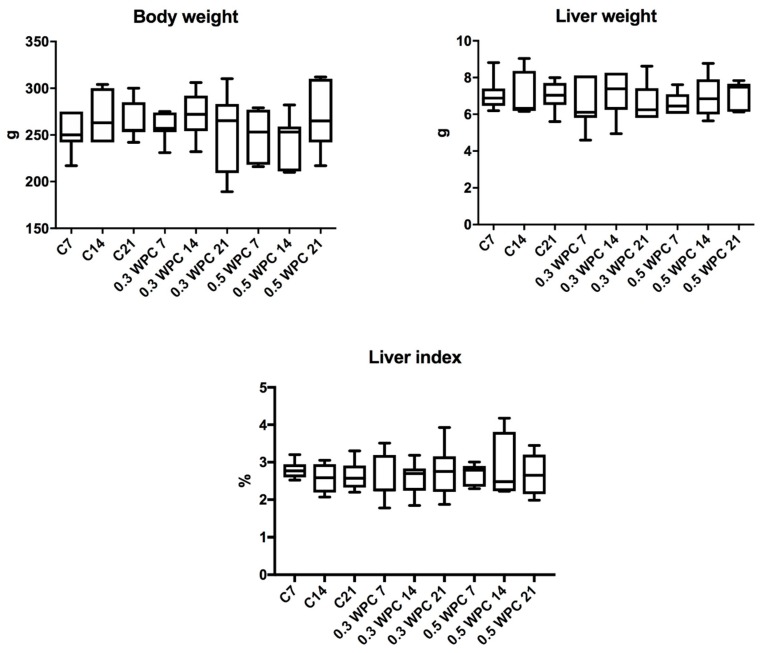
Body weight, liver weight and liver index of rats. C7, C14, C21, control groups; 0.3 WPC 7, 0.3 WPC 14, 0.3 WPC 21, 0.5 WPC 7, 0.5 WPC 14, 0.5 WPC 21, experimental groups.

No significant differences were found in the body weight, liver weight and liver index of the animals from different groups (Figure 1). Similarly, complete blood counts were not significantly different between the study group and control (Table 2).

Serum and plasma biochemical parameters did not differ significantly between the study and control rats (Table 3). Only serum ALT and AST level were statistically higher in the animals receiving WPC-80 at a dose of 0.5 g/kg body weight for 21 days versus control group (C21) (*p* = 0.0079; *p* = 0.0317 respectively). Similarly, only plasma creatinine and urea were significantly higher in the animals receiving higher dose of WPC-80 (0.5 g/kg body weight) for 21 days (*p* = 0.05; *p* = 0.0079) (Table 3).

**Figure 2:**
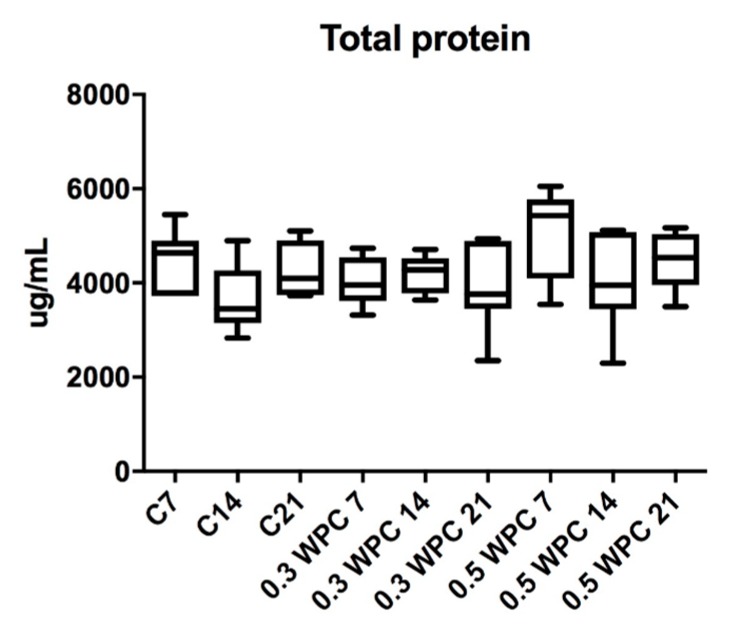
Total protein content in liver of rats fed with WPC-80 and the controls; C7, C14, C21, control groups; 0.3 WPC 7, 0.3 WPC 14, 0.3 WPC 21, 0.5 WPC 7, 0.5 WPC 14, 0.5 WPC 21, experimental groups.

The content of total protein in rat liver did not differ significantly between the study and control group rats (Figure 2).

### 3.3. Activity of Lysosomal Exoglycosidases

**Figure 3:**
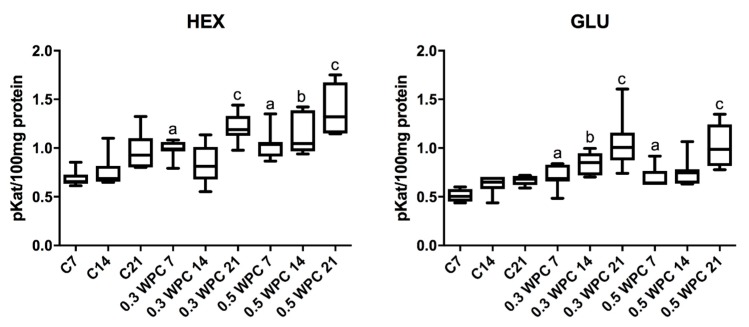
Activity of N-acetyl-β-D-hexosaminidase (HEX) and β-glucuronidase (GLU) in the liver of WPC-80-fed rats and the controls. C7, C14, C21, control groups; 0.3 WPC 7, 0.3 WPC 14, 0.3 WPC 21, 0.5 WPC 7, 0.5 WPC 14, 0.5 WPC 21, experimental groups. Differences statistically important: a vs C7; b vs C14; c vs C21 (*p* < 0.05).

Specific activity of HEX and GLU in liver homogenates of rats receiving WPC-80 at a dose of 0.3 and 0.5 g/kg body weight for 7 days was significantly higher compared to control group rats receiving physiological saline (C7) (HEX: 0.3 WPC 7 versus C7 *p* = 0.0012, 0.5 WPC 7 versus C7 *p* = 0.0006; GLU: 0.3 WPC 7 versus C7 *p* = 0.0700, 0.5 WPC 7 versus C7 *p* = 0.0210). GLU activity was considerably higher in the group supplemented with WPC-80 at a dose of 0.3 g/kg body weight for 14 days than in the control group (C14) (*p* = 0.011). Both HEX (*p* = 0.0006) and GLU (*p* = 0.0006) activity was significantly higher in the group of animals fed with whey at a dose of 0.3 g/kg body weight for 21 days (0.3 WPC 21) compared to WPC 7 and WPC 21 control groups and HEX activity in the group of 0.3 WPC 21 rats was considerably higher compared to the group receiving WPC-80 at the same dose for 14 days (0.3 WPC 14) (*p* = 0.0041) (Figure 3).

The specific activity of HEX in the group of rats receiving WPC-80 at a dose of 0.5 g/kg body weight for 14 days was significantly higher in comparison with both the control group (C14) (*p* = 0.0070) and group supplemented with a lower dose of whey (0.3 WPC 14) (*p* = 0.0175). The activity of HEX and GLU in the group of animals receiving WPC-80 supplementation at a dose of 0.5 g/kg body weight for 21 days was by far higher than in the 21-day control group (*p* = 0.0070, *p* = 0.0006 respectively). Similar changes were observed in the group of rats supplemented with the same dose of WPC-80 for 7 days. The specific activity of GLU in the group of 0.5 WPC 21 was also significantly higher compared to the group of animals fed with the same dose of the supplement for 14 days (*p* = 0.011) (0.5 WPC 14) (Figure 3).

**Figure 4:**
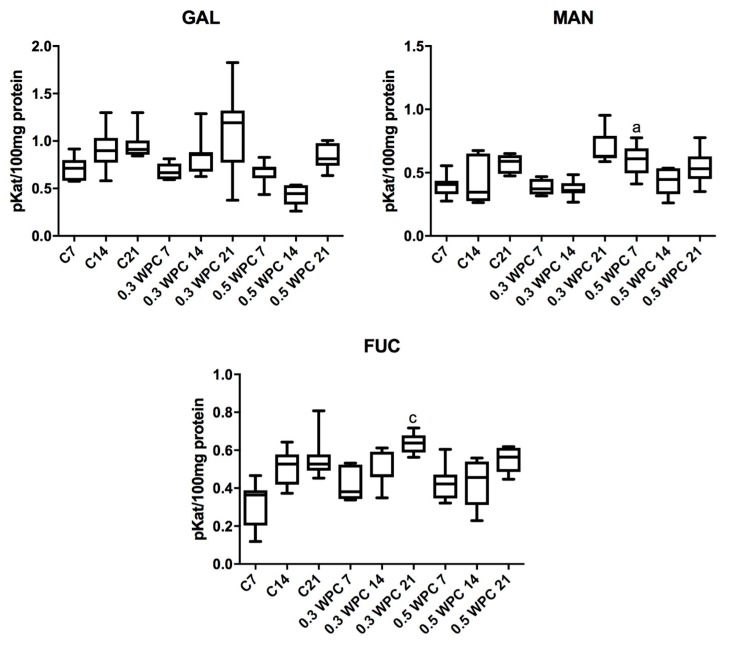
Activity of β-galactosidase (GAL), α-mannosidase (MAN) and α-fucosidase (FUC) in the liver of WPC-80-supplemented rats and the control group. C7, C14, C21, control groups; 0.3 WPC 7, 0.3 WPC 14, 0.3 WPC 21, 0.5 WPC 7, 0.5 WPC 14, 0.5 WPC 21, experimental groups. Differences statistically important: a vs C7; c vs C21 (*p* < 0.05).

The specific activity of GAL was significantly higher in the liver of rats fed with WPC-80 at a dose of 0.3 g/kg body weight for 21 days compared to the group receiving whey for 7 days (0.3 WPC 7) (*p* = 0.0379). Similar relation has been observed in rats receiving the supplement at a dose of 0.5 g/kg body weight: GAL activity was considerably higher in animals fed with WPC-80 for 21 days compared to those supplemented for 7 days (*p* = 0.0379) (Figure 4).

The specific activity of MAN in the liver of rodents fed with WPC-80 at a dose of 0.3 g/kg body weight for 21 days was significantly higher than in those receiving the same supplementation both for 7 (*p* = 0.0060) and 14 days (*p* = 0.0060) (Figure 4).

We demonstrated a significantly higher MAN activity in the liver of rats supplemented with WPC-80 at a dose of 0.5 g/kg body weight for 7 days (0.5 WPC 7) compared to the control group (C7) (*p* = 0.0041). Similarly, a considerably higher activity of the enzyme was observed in the group 0.5 WPC 7 compared to animals receiving WPC-80 at a dose of 0.3 g/kg body weight for the same period (*p* = 0.0041). In the group of rats receiving WPC-80 at a dose of 0.5 g/kg body weight for 14 days we observed significantly lower MAN activity than in the group fed with WPC-80 at the same dose for 7 days (*p* = 0.0379) (Figure 4).

The specific activity of FUC was significantly higher in the group of rats receiving WPC-80 at a dose of 0.3 g/kg body weight for 21 days compared to the control group (C21) (*p* = 0.0379) and the rats supplemented with WPC-80 at the same dose for 7 (*p* = 0.0060) and 14 (*p* = 0.011) days (Figure 4).

The specific activity of FUC was notably lower in the liver of rats fed with whey for 21 days at a dose of 0.5 g/kg body weight compared to the group of animals supplemented with WPC-80 at a dose of 0.3 g/kg body weight for 21 days. A significantly higher activity of the enzyme was also observed in the WPC-80-supplemented group receiving a dose of 0.5 g/kg body weight for 21 days than in the animals fed the same dose of the concentrate for 7 (*p* = 0.0175) and 14 days (*p* = 0.0379) (Figure 4).

### 3.4. Oxidative stress Markers

**Figure 5:**
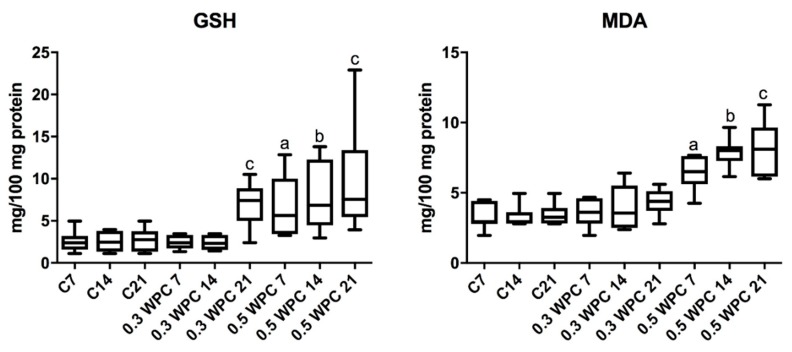
Reduced glutathione (GSH) and malondialdehyde (MDA) concentrations in the liver of WPC-80-fed rats and the control group. C7, C14, C21, control groups; 0.3 WPC 7, 0.3 WPC 14, 0.3 WPC 21, 0.5 WPC 7, 0.5 WPC 14, 0.5 WPC 21, experimental groups. Differences statistically important: a vs C7; b vs C14; c vs C21 (*p* < 0.05).

Significantly higher GSH concentration was obtained in the group of rats receiving WPC-80 at a dose of 0.3 g/kg body weight compared to the 21-day control group (*p* = 0.0260) and the group receiving whey at a dose of 0.3 g/kg body weight for 7 (*p* = 0.0152) and 14 (*p* = 0.0152) days (Figure 5).

The concentration of GSH was considerably higher in the group supplemented with WPC-80 at a dose of 0.5 g/kg body weight for 7 days in comparison with the controls (C7) (*p* = 0.0152). In the group of animals receiving WPC-80 at a dose of 0.5 g/kg body weight for 14 days we observed significantly higher concentration of GSH compared to both the group supplemented with a lower dose of whey (*p* = 0.0087) and the control group (C14) (*p* = 0.0152). Whey supplementation at a dose of 0.5 g/kg body weight for 21 days resulted in a significant increase in the concentration of GSH compared to the 21-day control group (*p* = 0.0043) (Figure 5).

The concentration of MDA was significantly higher in the group receiving 0.5 g/kg body weight of whey for 7 days compared to the control group (C7) (*p* = 0.0023) and the group receiving a lower dose of the supplement for the same period of time (0.3 WPC 7) (*p* = 0.0041). Significantly higher concentrations of malondialdehyde were observed in animals fed WPC-80 at a dose of 0.5 g/kg body weight for 14 days compared to both the control group (*p* = 0.0006) and the rats receiving 0.3 g/kg body weight of whey for 14 (*p* = 0.0012). The concentration of MDA was considerably higher in liver homogenates of rats receiving 0.5 g/kg body weight of WPC-80 for 21 days compared to the control group (*p* = 0.0006) as well as the rodents fed a lower dose of WPC-80 for 21 days (*p* = 0.0006) (Figure 5).

### 3.5. Pro-inflammatory Cytokines

**Figure 6:**
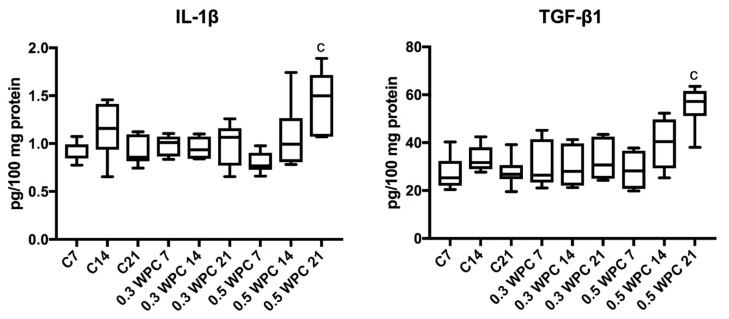
IL-1β and TGF-β1 levels in the liver of WPC-80-fed rats and the control group. C7, C14, C21, control groups; 0.3 WPC 7, 0.3 WPC 14, 0.3 WPC 21, 0.5 WPC 7, 0.5 WPC 14, 0.5 WPC 21, experimental groups. IL-1β, interleukin-1β; TGF-β1, transforming growth factor-β1. Differences statistically important: c vs C21 (*p* < 0.05).

The level of pro-inflammatory cytokines did not change between individual groups of rats. Only IL-1β and TGF-β1 levels were significantly higher in the liver of rats receiving WPC-80 at a dose of 0.5 g/kg body weight for 21 days (*p* = 0.0070; *p* = 0.0012 respectively) (Figure 6).

### 3.6. Correlations

The results of statistically significant correlations between the activity of exoglycosidases and oxidative stress parameters are presented in Table 4. Importantly, in the group of rats supplemented with WPC-80 at a dose of 0.3 g/kg body weight for 14 days we observed a positive correlation between MAN activity and MDA concentration. A similar correlation between GLU and MDA was observed in the group of rats fed the whey concentrate at a dose of 0.5 g/kg body weight for 7 days. In the same group of animals (0.5 WPC 7) a positive correlation between HEX activity and MDA concentration was also observed. In addition, a positive correlation was found between GLU and IL-1β in the groups of rodents receiving WPC-80 at a dose of 0.5 g/kg body weight for 14 (0.5 WPC 14) and 21 days (0.5 WPC 21). Serum ALT correlated with HEX and GLU in the group of rats supplemented with WPC-80 at a dose of 0.5 g/kg body weight for 21 days. In the same group of animals (0.5 WPC 21) a positive correlation between HEX and AST was also observed (Table 4).

### 3.7. Histological Examination

**Figure 7:**
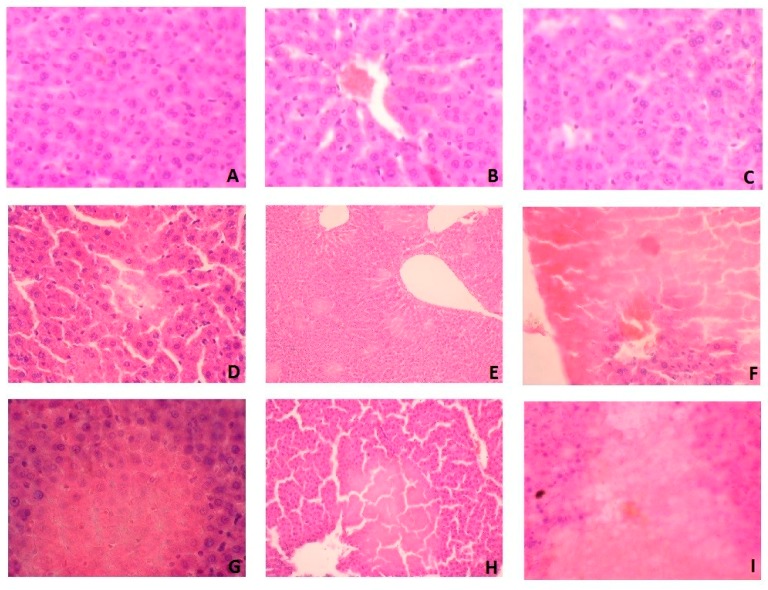
Liver histological examination of WPC-80-fed rats and the control group. **A** (C7), **B** (C14), **C** (C21), **D** (0.3 WPC 7), **E** (0.3 WPC 14), **F** (0.3 WPC 21), **G** (0.5 WPC 7), **H** (0.5 WPC 14), **I** (0.5 WPC 21).

In histological studies, we showed that WPC-80 leads to liver damage with the observed features of ischemic necrosis (Figure 7). In the liver of rats fed with WPC-80 at a dose of 0.3 g/kg body weight for 7 days, damage to single cells were demonstrated (Figure 7D). These changes were more pronounced in the liver of rats fed with WPC-80 at a dose of 0.5 g/kg body weight (0.5 WPC 7) (Figure 7G).

Feeding rats with WPC-80 at a dose of 0.3 g/kg body weight for 14 days caused focal lesions suggesting ischemic damage (Figure 7E), while increase of WPC-80 dose intensified these changes (Figure 7H). In addition, histological examination revealed the death of individual cell nuclei. In rats fed with a higher dose of whey protein concentrate (0.5 WPC 14), an increase in ischemic necrosis was observed by approx. 20% in relation to the dose of 0.3 g/kg body weight (0.3 WPC 14) (Figure 7E,H).

In rats fed with WPC-80 for 21 days at a dose of 0.3 g/kg body weight, hepatocellular damage was demonstrated with a loss of nuclei and obliteration of cell membranes (Figure 7F). More extensive changes were observed in the rats fed with a higher dose of WPC-80 (0.5 WPC 21) (Figure 7I). However, no signs of inflammation and liver fibrosis were confirmed in the histological examination (Figure 7).

## 4. Discussion

Our study is the first to indicate increased catabolism of liver glycoconjugates in the rats supplemented with whey protein concentrate WPC-80. We observed significantly higher activity of most lysosomal exoglycosidases as well as increased GSH concentration in the liver of rats receiving WPC-80. Despite increased glutathione biosynthesis, whey induces oxidative stress, which may lead to liver tissue damage.

Lysosomal exoglycosidases are responsible for the decomposition of sugar chains of glycoconjugates, that is, glycoproteins, glycolipids and proteoglycans in cell membranes and extracellular matrix [9,12]. Thus, changes in exoglycosidases activity may indicate catabolism and degree of remodelling of tissues/extracellular matrix [9]. Increased activity of exoglycosidases was recorded in the course of metabolic diseases [31], autoimmune diseases [32], cancer [33] and liver diseases [34,35,36]. The activity of exoglycosidases may be modified by environmental factors, diet and lifestyle [9]. Recently, a diet with high protein content (over 30% of the total energy intake) is becoming increasingly popular [37]. Excessive supply of dietary protein may affect the function of organs involved in protein metabolism (liver) and excretion of the products of its metabolism (kidneys) [7]. This condition leads to increased degradation of proteins due to the activity of, inter alia, hydrolytic lysosomal enzymes [7]. Therefore, changes in lysosomal exoglycosidases activity may reflect the intensity of metabolic changes occurring in liver tissue.

The liver is the main ‘metabolic organ’ of the human body. Its primary functions include decomposition of endogenous substances and xenobiotics (like toxins, alcohol and medicines), conversion of toxic ammonia into urea (in the so-called urea cycle) and participation in the metabolism of proteins, carbohydrates and lipids [7]. The results of our research indicate increased degradation of oligosaccharide chains of glycoconjugates in the liver of rats supplemented with WPC-80 concentrate. It is well known that hepatic glycoconjugates include structural, transport and secretory proteins, as well as tissue compatibility (histocompatibility) antigens encoded by MHC genes. The oligosaccharide chains of hepatic glycoproteins are also found on the outer surface of cell membrane, which participate in signal transduction and immune response pathways, while the glycoprotein transporters are involved in the transport of bile acids, glucose and growth factors [34,35,36,38]. It is suggested that increased catabolism of hepatic glycoconjugates may affect the cell structure and organisation of the liver tissue [39]. We found significant increase in the activity of most of the studied lysosomal hydrolases (↑HEX, ↑GLU, ↑MAN, ↑FUC) resulting from prolonged WPC-80 administration. However, the activity of GAL, GLU and partly HEX, FUC and MAN does not depend on the supplemented whey dose. Only the activity of HEX increased after two weeks of WPC-80 administration at a dose of 0.5 g/kg body weight compared to the dose of 0.3 g/kg body weight. It appears that raised activity of lysosomal hydrolases may indicate intensified liver metabolic processes and also impairment of liver function as a result of WPC-80 supplementation. Increased activity of exoglycosidases has been reported in numerous liver diseases, such as: primary biliary cirrhosis (PBC), autoimmune hepatitis (AIH), non-alcoholic fatty liver disease (NAFLD) and hepatic cholestasis [34,35,36,38]. It has been demonstrated that in inflammatory conditions lysosomal exoglycosidases are released from the liver into the blood, where they are caught by specific receptors located on the surface of macrophages [39]. In our study, the cytokines (IL-1β, TGF-β1) level was significantly higher in the liver of animals receiving WPC-80 at a dose of 0.5 g/kg body weight for 21 days (the highest dose and time of WPC-80 administration). It should be noted that TGF-β1 (transforming growth factor-β1) is one of the most important cytokines involved in the liver fibrosis [40]. It participates in the apoptosis of hepatocytes and activates inflammatory cells at the site of liver tissue damage. It is also the strongest factor stimulating the production of collagen and other extracellular matrix components [40]. Therefore, higher concentrations of TGF-β1 may predispose to the liver fibrosis in rats supplemented with high doses of WPC-80. Additionally, we observed a significant increase in GLU activity in the liver of WPC-80-supplemented rats compared to the controls. It is known that the activity of GLU increases in the course of numerous diseases of inflammatory aetiology [9]. This enzyme is released from the granular leukocytes and is one of the markers of the influx of neutrophils [41,42]. Numerous studies have described the relationship between GLU activity and concentration of proinflammatory cytokines and other inflammatory markers (e.g., IL-1, IL-6, TNF-α and CRP) [42,43]. Also in our experiment, the activity of GLU correlated with IL-1β level. Therefore, it may indicate intensified inflammatory processes in the liver parenchyma depending on the dose and time of administration of WPC-80. However, in the histopathological examination we did not observe any features of inflammation in the WPC-80-supplemented rats. Bearing in mind lysosomal exoglycosidases are an early marker of the inflammatory processes [42,43], an increase in their activity may suggest an initial stage of inflammation despite no changes were found in the histological studies. However, in WPC-80-fed rats we showed hepatocellular damage with the observed features of ischemic necrosis. Morphological changes of hepatocytes depended mainly on the duration of WPC-80 administration. Supplementation of WPC-80 at a dose of 0.5 g/kg body weight for 21 days resulted in a loss of nuclei as well as obliteration of cell membranes of the hepatocytes. Under such conditions, liver lysosomes may be damaged and proteolytic enzymes (including exoglycosidases) may be released. This may lead to further liver injury.

Oxidative stress may be one of the mechanisms responsible for the increase in the exoglycosidases activity in the liver. It is believed that a diet containing more than 33–45% protein may lead to increased production of ROS and initiate oxidative stress in various organs [18,20]. It is postulated that the increased production of free radicals is primarily a result of disorders of energy process in the mitochondrial respiratory chain [44]. In addition, it has been demonstrated that a long-term high-protein diet causes significant biochemical and ultrastructural changes in rat liver mitochondria and thus increase ROS production [45]. In our study, we found an increase of the MDA concentration in the liver of rats receiving WPC-80 at a dose of 0.5 g/kg of body weight compared to the control group. MDA is a marker of cell membrane lipid oxidation, which indicates oxidative stress in rats fed with WPC-80. MDA is also known to be a mutagenic and carcinogenic compound [46]. By forming adducts with proteins and nucleic acids, it promotes their accumulation in tissues and causes further oxidative damage [46,47]. MDA is also one of key oxidative stress mediators leading to liver damage through inflammation and fibrosis [48]. Lipid peroxidation may also cause necrosis of hepatocytes [46], which may partly explain the results of our histological studies. It is known that during hepatic ischemia, mitochondrial damage dramatically increases the ROS production, which intensifies further oxidative damage [49]. However, in our study we also observed an increase in the concentration of reduced glutathione—the most important liver antioxidant. This is not surprising as WPC-80 is a rich source of cysteine and methionine—precursors to GSH biosynthesis [3]. Yet, despite increased concentration of GSH in the group of rats receiving WPC-80, oxidative liver damage (↑MDA) occurs under these conditions. Therefore, the increased supply of WPC-80 does not effectively protect against excessive production of free oxygen radicals under the influence of increased protein supply. Moreover, it can be assumed that intensified GSH production is also an adaptive response of the body to increased ROS production, especially at high doses of WPC-80. Positive correlation between MDA and lysosomal exoglycosidases (HEX, GLU and MAN) in the liver of WPC-80-supplemented rats may prove the potential relationship between oxidative stress and catabolism of glycoconjugates, as it is suggested that oxidative stress increases permeability of lysosomal membranes and leakage of lysosomal exoglycosidases into the bloodstream.

In the literature, there are only a few studies evaluating the influence of a high-protein diet on the profile of lysosomal exoglycosidases in the liver. Witek et al. [50] analysed the activity of selected hydrolases (HEX, GLU, GAL, acid phosphatase, leucine and alanine aminopeptidase, cathepsins D and L) in the liver and kidneys of mice receiving a diet of varied (10% and 16%) protein content. A significant increase in lysosomal exoglycosidases activity as well as a considerable decrease in aminopeptidase and cathepsin activity was found in the liver of rats fed on a higher protein content diet. However, the reasons for the observed changes are still unknown. Colombo et al. [51] demonstrated significantly higher activity of AST, ALT and GGT in the liver of rats receiving a diet containing 32% and 51% protein versus the controls. Moreover, Mutlu et al. [52] reported increased activity of hepatic aminotransferases, as well as increased concentration of albumins with concomitant abdominal pain, which may suggest the need for caution in case of a high-protein diet. In our study, we showed increased aminotransferases activity in rats receiving WPC-80 at a dose of 0.5 g/kg body weight versus control group. Importantly, serum ALT and AST correlated with the activity of hepatic exoglycosidases (HEX, GLU), further confirming the usefulness of lysosomal hydrolases as markers of the liver function.

We did not observe any significant changes in the body weight, liver weight and liver index of rats receiving WPC-80 compared to controls. Although a high-protein diet is known to regulate the fat deposition in the liver [53], in our research, whey protein concentrate was not the primary source of food. It was administered as the intragastric supplementation, which may explain the observed lack of differences. However, in addition to the liver, also kidneys play a major role in the metabolism of proteins. Although this was not a direct goal of the present study, it may be presumed that high WPC-80 doses may interfere with kidney function as evidenced by the increase in serum creatinine and urea. However, this assumption requires further research especially because the remaining biochemical (albumin, uric acid) and morphological parameters did not differ significantly between individual groups of rats.

To sum up, WPC-80 is responsible for intensified reconstruction of liver tissue and induction of oxidative stress. We observed a significant increase in the activity of most lysosomal exoglycosidases and raised oxidative damage compared to the controls. In addition, whey protein concentrate leads to hepatocellular damage with the observed features of ischemic necrosis. High activity of exoglycosidases may result from their participation in liver tissue remodelling or involvement in an ongoing inflammatory process, particularly under the influence of high doses of WPC-80 (0.5 g/kg body weight). Since our experiment does not explain the reasons for the observed changes, we would like to highlight the need of conducting further studies to assess the effect of WPC-80 on liver damage parameters. We believe that it is particularly important to properly select the dose and time of WPC-80 administration.

Finally, it is also worth noting that our work had certain limitations. We evaluated only the selected lysosomal enzymes and biomarkers of oxidative stress and inflammation, so we cannot fully characterize the catabolism of liver glycoconjugates or changes caused by free radicals as a result of WPC-80 supplementation. What is more, our study assessed an animal model that can never fully replace tests on humans. However, despite relatively many reports on the antioxidant properties of whey, our study is the first to indicate the induction of liver oxidative stress under the influence of whey protein concentrate. We also point out that WPC-80 enhances catabolism of liver glycoconjugates and changes the morphology of hepatocytes. Therefore, whey should be cautiously used in liver diseases and disorders of the gastrointestinal tract. Our research is also a starting point for future basic and clinical research.

## Figures and Tables

**Table 1:** General composition of whey protein concentrate (WPC-80).

General Composition	%
Protein	78.2
Fat	6.72
Carbohydrates	7.9
Ash	2.7
Dietary fibre	<0.5
Humidity	4.5
The calorific value	1712 kJ/100 g

**Table 2:** Complete blood counts in rats fed with WPC-80 and the controls [median (minimum-maximum)].

	WBC (× 10^12^/L)	RBC (M/μL)	HGB (g/dL)	HTC (%)	MCV (fL)	MCH (pg)	MCHC (g/dL)	PLT (× 10^9^/L)
C7	3.3 (2.8–3.7)	7.1 (6.6–9.9)	13.8 (13.6–13.9)	40.0 (38.9–43.0)	58.5 (58.0–59.0)	20.5 (18.3–21.1)	35.2 (34.8–35.6)	781.5 (635.0–928.0)
C14	2.9 (2.5–3.8)	7.0 (6.7–7.4)	14.0 (12.8–15.0)	41.6 (39.4–45.2)	59.0 (57.0–62.00)	19.9 (19.0–21.2)	33.2 (31.1–34.5)	825.0 (747.0–881.0)
C21	3.5 (2.5–4.8)	7.4 (6.8–8.9)	15.3 (12.3–17.4)	49.2 (36.2–52.2)	58.5 (56.0–59.0)	19.5 (18.2–20.5)	33.2 (31.1–33.3)	713.5 (406.0–821.0)
0.3 WPC 7	3.6 (2.8–4.9)	7.3 (6.3–7.9)	14.9 (12.9–15.8)	43.0 (26.5–46.0)	58.5 (57.0–59.0)	20.2 (19.8–20.8)	34.7 (34.4–35.4)	751.5 (627.0–914.0)
0.3 WPC 14	3.3 (2.4–4.0)	7.1 (6.4–8.1)	14.1 (12.7–15.3)	42.1 (37.2–44.8)	57.5 (54.0–62.0)	19.6 (18.0–20.5)	34.2 (31.8–34.7)	743.5 (462.0–920.0)
0.3 WPC 21	3.2 (2.5–4.8)	7.4 (6.5–8.7)	14.8 (13.2–16.4)	42.6 (36.7–49.5)	58.0 (55.0–60.0)	20.0 (18.0–21.0)	35.1 (33.0–36.4)	751.0 (667.0–855.0)
0.5 WPC 7	3.9 (3.2–5.0)	6.9 (6.4–8.9)	14.2 (13.0–17.8)	40.9 (37.3–50.8)	58.0 (57.0–61.0)	20.4 (20.0–20.6)	35.0 (33.8–35.7)	760.0 (589.0–930.0)
0.5 WPC 14	3.2 (2.6–3.9)	7.5 (6.6–8.0)	13.9 (13.3–16.0)	44.0 (39.8–54.0)	59.0 (42.9–60.0)	19.8 (14.2–20.0)	33.5 (18.0–35.6)	720.0 (331.0–778.0)
0.5 WPC 21	3.7 (2.8–5.4)	7.8 (6.6–8.9)	15.1 (13.7–16.6)	45.0 (38.7–49.7)	57.5 (50.0–61.0)	19.8 (17.9–20.9)	33.7 (32.0–35.3)	709.5 (599.0–880.0)

C7, C14, C21, control groups; 0.3 WPC 7, 0.3 WPC 14, 0.3 WPC 21, 0.5 WPC 7, 0.5 WPC 14, 0.5 WPC 21, experimental groups. HCT, haematocrit; HGB, haemoglobin; MCH, Mean Corpuscular Haemoglobin; MCHC, Mean Corpuscular Haemoglobin Concentration; MCV, Mean Corpuscular Volume; PLT, platelets; RBC, erythrocytes; WBC, leukocytes.

**Table 3:** Serum and plasma biochemical parameters in rats fed with WPC-80 and the controls [median (minimum-maximum)].

	ALT (U/L)	AST (U/L)	Bilirubin (mg/dL)	Albumin (μmol/L)	Cr (mg/dL)	UA (μmol/L)	Urea (mmol/L)	TC (mmol/L)	Glucose (mg/dL)
C7	52.6 (34.1–64.7)	100.3 (65.9–133.1)	1.6 (1.5–1.7)	419.9 (416.5–432.8)	0.5 (0.4–0.6)	27.0 (26.0–36.0)	6.4 (4.3–6.7)	80.7 (61.2–98.7)	94.2 (68.0–102.5)
C14	52.0 (42.8–66.6)	88.0 (76.0–117.7)	1.7 (1.0–2.0)	420.4 (337.0–435.8)	0.4 (0.4–0.5)	27.0 (24.0–34.0)	6.3 (5.6–8.6)	83.3 (51.3–97.0)	89.0 (83.0–92.0)
C21	47.2 (38.1–73.8)	91.5 (75.9–116.8)	2.0 (1.8–2.3)	417.2 (385.3–435.4)	0.5 (0.4–0.7)	26.0 (16.0–34.0)	8.2 (6.5–8.5)	84.0 (78.6–95.6)	89.0 (71.3–100.0)
0.3 WPC 7	59.0 (35.5–88.4)	122.2 (93.6–132.4)	1.2 (1.0–1.3)	418.7 (368.2–447.8)	0.4 (0.3–0.5)	24.0 (17.0–43.0)	5.8 (4.6–6.1)	70.4 (40.9–98.5)	89.0 (78.0–104.0)
0.3 WPC 14	55.2 (50.8–61.6)	115.1 (106.0–135.9)	1.1 (0.9–1.9)	461.3 (432.3–500.8)	0.5 (0.4–0.6)	29.0 (24.0–45.0)	7.8 (5.8–8.6)	88.7 (62.7–101.4)	85.0 (72.0–105.5)
0.3 WPC 21	57.0 (49.1–76.8)	103.3 (80.0–141.0)	1.2 (0.9–2.0)	419.7 (384.0–471.6)	0.5 (0.5–0.6)	36.0 (27.0–39.0)	8.1 (7.2–9.2)	90.1 (69.1–95.0)	82.5 (68.0–96.00)
0.5 WPC 7	57.5 (42.7–81.2)	86.0 (73.0–158.7)	2.0 (1.6–2.4)	460.4 (445.3–486.4)	0.6 (0.5–0.8)	29.0 (21.0–45.0)	6.0 (5.3–7.2)	79.7 (63.7–82.1)	90.5 (83.0–97.0)
0.5 WPC 14	57.9 (49.6–99.8)	109.4 (107.6–148.4)	1.7 (1.4–2.1)	430.0 (401.7–442.7)	0.5 (0.5–0.7)	33.0 (24.0–45.0)	6.9 (5.7–7.8)	88.8 (70.3–89.7)	78.0 (74.0–98.00)
0.5 WPC 21	101.1 (77.5–126.0)^c^	173.0 (164.6–216.2)^c^	1.3 (0.8–2.4)	464.7 (388.6–469.7)	0.7 (06–0.9)^c^	33.0 (23.0–36.0)	9.7 (8.9–10.4)^c^	78.0 (58.4–95.2)	85.5 (73.0–101.0)

C7, C14, C21, control groups; 0.3 WPC 7, 0.3 WPC 14, 0.3 WPC 21, 0.5 WPC 7, 0.5 WPC 14, 0.5 WPC 21, experimental groups. ALT, alanine aminotransferase; AST, aspartate aminotransferase; Cr, creatinine; TC, total cholesterol; UA, uric acid. Differences statistically important: c vs C21 (*p* < 0.05).

**Table 4:** Correlations between the profile of lysosomal exoglycosidases, oxidative stress and biochemical parameters in rats fed with WPC-80.

Pair of Variable	Group	*r*	*p*
MAN & MDA	0.3 WPC 14	0.886	0.003
GLU & MDA	0.5 WPC 7	0.812	0.005
HEX & MDA	0.5 WPC 7	0.607	0.05
HEX & Cr	0.5 WPC 7	0.650	0.05
GLU & GSH	0.5 WPC 14	0.771	0.04
GLU & IL-1β	0.5 WPC 14	0.650	0.05
GLU & IL-1β	0.5 WPC 21	0.810	0.005
HEX & ALT	0.5 WPC 21	0.741	0.001
HEX & AST	0.5 WPC 21	0.690	0.04
GLU & ALT	0.5 WPC 21	0.920	<0.0001

0.3 WPC 14, 0.5 WPC 7, 0.5 WPC 14, experimental groups; ALT, alanine aminotransferase; AST, aspartate aminotransferase; Cr, creatinine; GLU, β-glucuronidase; GSH, reduced glutathione; HEX, N-acetyl-β-d-hexosaminidase; IL-1β, interleukin-1β; MAN, α-mannosidase; MDA, malondialdehyde; MAN, α-mannosidase.

## Supplementary Materials

The following are available online at http://www.mdpi.com/2072-6643/10/9/1178/s1, Table S1: Protein and amino acids in analysed WPC-80., Table S2: Fatty acids in analysed WPC-80. Table S3: Vitamins and minerals in analysed WPC-80.
