# Supplementary material for: Whey Protein Concentrate WPC-80 Intensifies Glycoconjugate Catabolism and Induces Oxidative Stress in the Liver of Rats
Source: Nutrients. 2018 Aug 28;10(9):1178. doi: 10.3390/nu10091178 (PMC6164859; doi:10.3390/nu10091178)
Supplement: Supplementary file 1 [file nutrients-10-01178-s001.pdf]

**Protein**	%
β-lactoglobulin	41.27
α-lactalbumin	10.92
immunoglobulins	6.56
bovine serum albumin	3.75
**Endogenous amino acids**	%
aspartic acid	9.09
glutamic acid	15.02
serine	4.41
glycine	1.58
tyrosine	2.73
arginine	2.24
cysteine	1.62
alanine	4.11
proline	5.23
**Exogenous amino acids**	%
histidine	1.43
valine	5.07
methionine	1.97
threonine	5.99
isoleucine	4.91
leucine	9.04
phenylalanine	2.90
lysine	7.96
tryptophan	13.04 g/kg

Table 1. Protein and amino acids in analyzed WPC-80.

**Fatty acids**	**g/100g fat**
butanoic acid (butyric acid) C4:0	1.7
hexanoic acid (caproic) C6:0	0.9
octanoic acid (caprylic) C8:0	0.8
decanoic acid (capric) C10:0	2.2
undecanoic acid C11:0	<0.1
dodecanoic acid (lauric) C12:0	2.9
tridecanoic acid C13:0	< 0.1
tetradecanoic acid (myristic) C14:0	11.4
pentadecanoic acid C15:0	1.2
hexadecanoic acid (palmitic) C16:0	33.9
heptadecanoic acid C17:0	0.7
octadecanoic acid (stearic) C18:0	11.0
eicosanoic acid (arachidic) C20:0	0.2
docosanoic acid (behenic acid) C22:0	0.3
tetracosanoic acid C24:0	0.2
tetradecenoic acid C14:1	0.9
pentadecenoic acid C15:1	< 0.1
haxadecenoic acid (palmitoleic) C16:1	2.2
heptadecenoicacid C17:1	0.2
(Z) -9-Octadecenoic acid (Oleic) C18:1	24.1
eicosenoic acid(gadoleic) C20:1	<0.1
(Z) -13-docozaenowy acid C22:1	< 0.1
nervonic acid C24:1	< 0.1
(Z, Z) -9.12-octadecadienoic C18:2	1.5
octadecatrienoic acid C18:3	0.3
eicosadienoic acid C20:2 n-6	< 0.1

docosadienoic acid C22:2 n-6	< 0.1
other fatty acids	3.4

Table 2. Fatty acids in analyzed WPC-80.

**Vitamins**	**per 100g**
Vitamin A (retinol)	20 μg
Vitamin B1 (thiamine)	0.11 mg
Vitamin B2 (riboflavin)	1.0 mg
Vitamin B6 (pyridoxine)	0.11 mg
Vitamin E (α-tocopherol)	0.1 mg
Vitamin B12	28.4 μg
Vitamin B9	621 μg
Vitamin D_3	<0.5 μg
Vitamin K_3	<1 mg/kg
**Minerals**	
sodium	0.15%
potassium	0.53%
calcium	0.46%
phosphorus	0.31%
magnesium	784 mg/kg
zinc	4.88 mg/kg
copper	0.98 mg/kg
iron	2.86 mg/kg

Table 3. Vitamins and minerals in analyzed WPC-80.
